# Symptom severity is associated with leftward lateralization upon contextual modulation of visual vertical in patients with schizophrenia

**DOI:** 10.3389/fpsyt.2022.948114

**Published:** 2022-07-18

**Authors:** Abdul Rima Razzak, Haitham Jahrami, Mariwan Husni, Maryam Ebrahim Ali, Jeff Bagust

**Affiliations:** ^1^Department of Physiology, College of Medicine and Medical Sciences (CMMS), Arabian Gulf University (AGU), Manama, Bahrain; ^2^Ministry of Health (MOH), Manama, Bahrain; ^3^Department of Psychiatry, College of Medicine and Medical Sciences (CMMS), Arabian Gulf University (AGU), Manama, Bahrain; ^4^Northern Ontario School of Medicine University, Ontario, ON, Canada; ^5^Faculty of Health and Social Sciences, Bournemouth University, Poole, United Kingdom

**Keywords:** context modulation, schizophrenia, lateralization, spatial orientation, negative

## Abstract

**Background:**

Contextual processing dysfunction in patients with schizophrenia (SCZ) is not uniform and task-dependent. In SCZ, studies on the rod and frame test (RFT), which evaluates contextual modulation of verticality perception, are sparse. A main study that utilized a two-alternative forced choice design for judging rod verticality reported equivalent strength of RFT contextual modulation in healthy controls and SCZ. The current study aims to uncover any potential differences in contextual modulation between controls and SCZ with an adjustment method on a computerized RFT.

**Materials and methods:**

A total of 17 healthy controls and 15 SCZ aligned an oriented rod to their perceived vertical with a computer mouse under four randomized frame presentations: absent frame, non-tilted (Frame^0°^), or tilted by 18 degrees leftward (Frame^–18°^) or rightward (Frame^+18°^). Rod deviation error was assigned a negative or positive value when aligned leftward or rightward, respectively, of 0°. Signed and absolute errors, the rod and frame effect (RFE), and intra-individual variability (inconsistency) were used for analysis.

**Results:**

There was no group difference in rod alignment errors or derived measures, except that SCZ displayed greater inconsistency in rod alignment, compared to controls. The negative symptom scale (PANSS-N) scores correlated positively with the variability measure and with unsigned Frame^–18°^ error.

**Conclusions:**

Only the variability measure was sensitive enough to distinguish between controls and SCZ. SCZ with more severe negative symptoms had larger variability in rod alignment, probably reflecting a state of indifference. The larger deviation errors only with a leftward tilted frame, as PANSS-N scores increased, may indicate a lateralized attentional abnormality that is correlated with severity of symptoms in SCZ.

## Introduction

Visual vertical representation is central to spatial perception, specifically to determining the orientation of lines and objects (1). However, the mechanisms by which the brain processes the orientation characteristics of an object can be markedly affected by surrounding contextual elements. For instance, the perceived orientation of a line is affected by the orientation structure of the surrounding image. This can be assessed by the rod and frame test (RFT) which evaluates contextual modulation of verticality perception, as the orientation of a central rod has to be judged while it is surrounded by a larger oriented frame (2). For most observers, the judgment of the orientation of the individual rod is influenced by the surrounding context, with illusions occurring in the direction of frame tilt, as observers place the rod toward the direction of the surrounding frame tilt. This is known as the rod and frame effect (RFE).

Perceptual contextual effects in vision have been widely used to study a range of human conditions, and several studies on schizophrenia patients (SCZ) have reported a broad deficit in visual contextual processing that is manifested in weaker contextual effects (3–5). On the other hand, there is strong evidence suggesting that abnormal contextual modulation in schizophrenia is task or domain selective (5), arguing against the proposed unitary contextual processing dysfunction in SCZ (5, 6). An important factor reported to determine the degree of deficits in visual contextual processing for SCZ is the sensory level of integration at which contextual modulations occur. SCZ usually exhibit great resistance mainly to higher-level integration contextual modulations, which usually require more complex cognitive operations in order to produce their effects (7).

The neuroanatomical substrates of visual vertical judgment are at a cortical level bilaterally (1). Furthermore, contextual effects that involve orientation judgments in general are considered to be predominantly cortically mediated (8). In line with this, weaker contextual modulation of visual vertical is to be expected in SCZ. To our knowledge, studies that have assessed the strength of contextual modulation of the orientation of a central rod on the RFT are sparse for SCZ. An exception is a previous study that has shown an equivalent strength of RFT contextual modulation in healthy controls and SCZ (9), indicating that the magnitude of modulations associated with orientation was similar between the two groups. Yet, that study was based on a two-alternative forced choice design for judging the verticality of the rod, where the participant’s task was to verbally report whether the rod was toward the left or the right side of the vertical position. It has been suggested that this kind of design is suboptimal for the purposes of measuring perception, and that manual adjustments should be used instead to try and reduce any confounding factors that forced-response tasks may introduce in contextual modulation research (7).

The current study aims to compare contextual modulation on the RFT between healthy control subjects and SCZ, as they adjust the rod to their perceived vertical with a computer mouse on a computerized version of the RFT. Unlike the two-alternative forced choice design, this adjustment procedure allows participants to demonstrate quantitatively how they perceive the verticality of the rod upon contextual modulation. We expect that the adjustment method along with enhanced computer-based recording resolution may be quite effective in detecting any potential group differences between healthy controls and SCZ. We also aim to explore any associations between the strength contextual modulation on the RFT and clinical characteristics or psychotic symptoms in SCZ, since severity of visual processing impairments, such as strong orientation context effects, are known to be correlated with worse symptoms and social functioning in these patients (10, 11).

## Materials and methods

### Participants

Fifteen patients (4 females, 27%) who met the Diagnostic and Statistical Manual of Mental Disorders 5th Edition (DSM-5) criteria for schizophrenia were recruited from the national center for diagnosis and treatment of severe mental illnesses in Bahrain. All diagnoses were made by a multi-disciplinary team led by a psychiatry consultant in Bahrain.

Simple random sampling was performed to obtain a sample that statistically represents the population. For patients, the business intelligence module in the national electronic medical record, the function “= RAND ()” was used to generate a pool of patients from which our subjects were at the top of the list. We generated a larger pool than anticipated to recruit because participants might have been unavailable for various reasons including travel, COVID-19 quarantine etc.

At the time of the study, all but one of the patients were on an atypical antipsychotic medication. All patients had a combination of positive and negative symptoms. All SCZ were medicated (93% on atypical antipsychotic drugs, 27% on typical antidepressants, and 27% taking both). None of the patients were on atypical or combination ant-depressant drugs, or on lithium and propranolol tranquilizers. Clinical symptoms were assessed with the Positive and Negative Syndrome Scale (PANSS) (12) by well-trained psychiatrists. PANSS is regarded as the “gold standard” by which all assessments of psychotic behavioral disorders should be judged. The overall score on the PANSS scale ranges from 30 to 210. The positive scale contains 7 items, (minimum score = 7, maximum score = 49). The items are delusions, conceptual disorganization, hallucinations, excitement, grandiosity, suspiciousness/persecution, and hostility. The negative scale has also 7 items, (minimum score = 7, maximum score = 49). The items are blunted affect, emotional withdrawal, poor rapport, passive/apathetic social withdrawal, difficulty in abstract thinking, lack of spontaneity and flow of conversation, stereotyped thinking. Finally, the general psychopathology scale has 16 items, (minimum score = 16, maximum score = 112). The items are somatic concern, anxiety, guilt feelings, tension, mannerisms and posturing, depression, motor retardation, uncooperativeness, unusual thought content, disorientation, poor attention, lack of judgment and insight, disturbance of volition, poor impulse control, preoccupation, active social avoidance. The PANSS demonstrated strong interrater and test–retest reliability (0.92 and 0.75, respectively).

All SCZ patients had both negative and positive symptoms, and experienced auditory hallucinations.

The participants did not have any visual hallucinations as part of their clinical symptoms profile.

This cohort of patients did not have any nystagmus, cataract, glaucoma as per their physical examination history and National Health records.

Seventeen healthy age and gender matched control subjects (5 females, 29%) with no history of mental illness, neurological, or ophthalmic disorders were recruited among the staff from the same psychiatry hospital. These healthy individuals were taken into a control group. No control subject was receiving any regular medications.

Exclusion criteria include important comorbid psychiatric disorder, neurological or medical disorders, severe visual loss, history of severe head trauma, alcohol/substance dependence or abuse, electro-convulsive therapy in recently initiated treatment for SCZ.

Approval for conducting this study was taken from the Secondary Health Care Research Committee (SHCRC) at the Ministry of Health (MOH) and form the Research and Ethics Committee at the College of Medicine and Medical Sciences at the Arabian Gulf University in Bahrain (Reference: E23-PI-01/20).

After the aim and impact of the study were explained to the healthy controls and to the patients and their relatives, informed consent was taken, and those who volunteered were included in the study. All participants were right-handed on self-report. Table 1 displays demographic characteristics for participants and clinical characteristics for the patient group.

**TABLE 1:** Demographic characteristics for all participants, with clinical characteristics for the SCZ.

	Control (*n* = 17)	SCZ (*n* = 15)	
**Age (years)**	32.65 ± 9.16 (20–53)	34.73 ± 14.87 (18.0–67.0)	*t* = 0.48, *P* = 0.63
**College education level**			
Primary	2 (12%)	0 (0%)	Chi- Square = 6.26
Secondary	5 (29%)	10 (67%)	*P* = 0.10
BSc	8 (47%)	5 (33%)	
Post-graduate	2 (12%)	0 (0%)	
Age at diagnosis of disease (years)		20.73 ± 4.40 (15.0–28.0)	
Duration of illness (years)		14.0 ± 11.36 (1.0–39.0)	
Inpatient stay duration (years)		3.87 ± 2.26 (1.0–8.0)	
**Scores on psychiatry tests**			
PANSS–N		24.07 ± 7.11 (9.0–32.0)	
PANSS–P		20.87 ± 7.04 (11.0–31.0)	
PANSS–GP		29.0 ± 8.03 (19.0–41.0)	

PANSS, the positive and negative syndrome scale. “N” for negative symptoms, “P” for positive symptoms, “GP” for general psychopathology subscale.

### Measurement of visual vertical: The computerized rod and frame test

**FIGURE 1:**
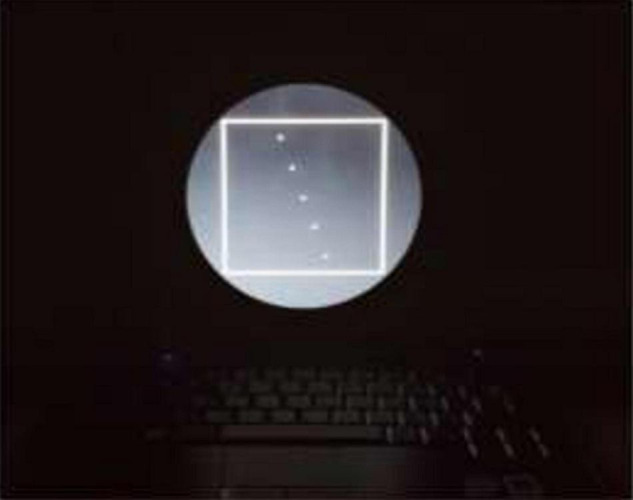
Concealment of the vertical edges of the laptop with a circular black paper ring stuck to the laptop screen.

We utilized a computerized version of the rod and frame test (CRFT) to assess verticality judgement (13). A virtual line marked by five white dots at its ends was used instead of a continuous line to reduce clues to verticality, which might be provided by the stepped appearance of a displayed solid line (14). The test was performed while sitting in a comfortable position with no head restraint; however, participants were instructed to keep their trunks and heads fixed and maintain their feet in a flat position. A round black paper ring was stuck on the laptop screen to conceal its edges and reduce clues to verticality, while exposing the rod and frame/disc presentation in the center of the screen (Figure 1). The test was performed in a dark room minimizing further any vertical cues within the room.

**FIGURE 2:**
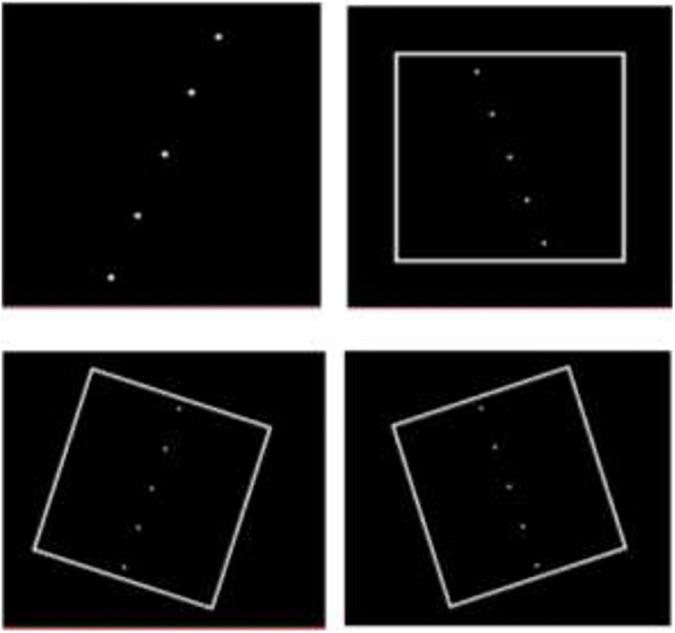
Presentations of “rod and frame” during testing. Some presentations were with no surrounding frame. For frame context, the frame was displayed in a randomized order as either erect or tilted by 18° clockwise or counter clockwise.

Participants rotated the dots around their virtual center in 0.5° increments in either clockwise (CW) or counter clockwise (CCW) directions using the mouse buttons until the “rod” was considered vertical. The space bar of the computer keyboard was then pressed to record the rod deviation relative to vertical and move the program to the next presentation. Recording of rod deviation tilt was conducted with a test sequence consisting of a series of 18 individual Rod and Frame presentations. In each case, the first two presentations were for subject training and were discarded in the analysis. The next 16 presentations were displayed in a randomized order and consisted of 4 presentations with no visual reference\frame [subjective visual vertical (SVV)] and 12 presentations in three visual frame contexts with four trials for each visual context: Non-tilted frame (0°, Frame^0°^); the frame tilted CCW (−18°, Frame^–18°^) or tilted CW (+18°, Frame^+18°^) with respect to the vertical (Figure 2).

Participants were informed of the importance of spatial accuracy, and there was no time restriction for completing the task.

### Computerized rod and frame test analysis

The angular deviation of the rod’s final position from true vertical was recorded as error in degrees. According to convention, CW tilts of the rod by the participants were denoted by a positive value, whereas CCW tilts were considered negative. Both unsigned (absolute) and signed (algebraic) values of deviation error were used in this study.

#### Intraindividual variability measure

An analysis was done to detect intraindividual variability about verticality judgement. This measure, calculated as the standard deviation of the signed or algebraic error, reflects the precision of the tilts in the roll plane. A large value of variability measure indicates that the participant is not responding consistently, or in other words not with precision. Due to the three different frame/disc conditions and two starting positions for rod (CCW −20° and CW 20°), there are six different combinations of rod and frame conditions used in the CRFT (Table 2).

**TABLE 2:** The six different combinations of rod and frame conditions.

Frame condition	Rod starting position	
	Counterclockwise (−20°)	Clockwise (+20°)
Frame^0°^	*q_1_, q_2_*	*r_1_, r_2_*
Frame ^–18°^	*u_1_, u_2_*	*v_1_, v_2_*
Frame ^+18°^	*w_1_, w_2_*	*x_1_, x_2_*

The subscripts 1 and 2 represent the trial number for the rod starting position.

For the six combinations representing the frame conditions (Frame^0°^, Frame^–18°^, and Frame^+18°^), the equation for the variability measure “σ°” is given by:


$$\begin{array}{c} \sigma =\text{SQRT}(\left({\left(q1-q2\right)}^{2}/2\right)+\left({\left(r1-r2\right)}^{2}/2\right)+\left({\left(u1-u2\right)}^{2}/2\right)\\ \;+\left({\left(v1-v2\right)}^{2}/2\right)+\left({\left(w1-w2\right)}^{2}/2\right)+\left(\left(\frac{x1-x2){}^{2}}{2}\right)\right)/6) \end{array}$$

#### Frame effects

Parameters derived from the algebraic errors include the RFE induced by the tilted frame around the untilted frame error. This provided a measure of the influence of the surrounding tilted frame on the judgement of vertical. These effects were calculated by:


$${\mathrm{RFE}}^{-18^{{}^{\circ}}}={\mathrm{Frame}}^{-18^{{}^{\circ}}}-{\mathrm{Frame}}^{0^{{}^{\circ}}}$$

$${\mathrm{RFE}}^{+18^{{}^{\circ}}}={\mathrm{Frame}}^{+18^{{}^{\circ}}}-{\mathrm{Frame}}^{0^{{}^{\circ}}}$$

### Statistical analysis

Analysis was performed by SPSS (Version 28). All verticality judgement data followed a normal distribution when evaluated with the Kolmogorov and Smirnov test, so parametric tests were used for analyses of group means. A Two-Way mixed ANOVA was used to determine the effect of psychotic condition (Control, Patient) and frame condition (Frame^0°^, Frame^–18°^, Frame^+18°^) on rod deviation errors. An unpaired *t*-test was used to compare between control and patient groups the SVV error, the intraindividual variability, and the RFE measure. Spearman Rank Order correlations were used to evaluate associations between any two variables for SCZ.

## Results

**FIGURE 3:**
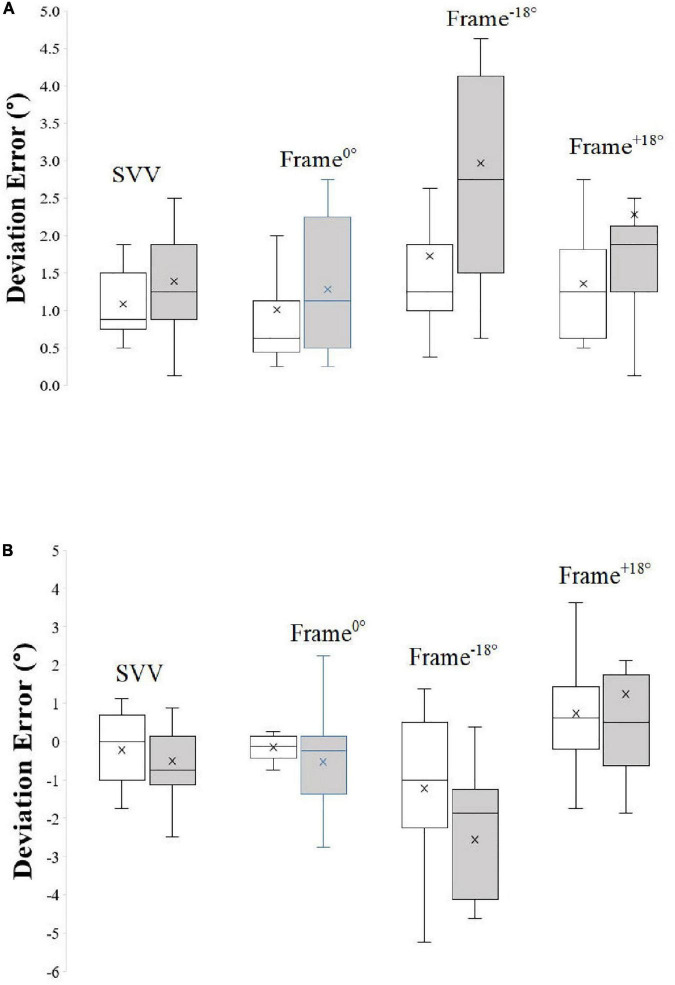
Box and Whisker plots of unsigned **(A)** and signed **(B)** deviation error with median and interquartile range during different frame contexts in control participants and SCZ. “X” represents the mean of the data.

Figure 3 depicts Box and Whisker plots of signed and unsigned deviation error with median and interquartile range during different frame contexts in control participants and SCZ.

### Absolute (Unsigned) error

There was no significant difference (*t* = 1.55, *P* = 0.13, df = 30) between SVV (no-context control condition) absolute error between controls (mean = 1.08 ± 0.43°) and SCZ (mean = 1.40 ± 0.69°).

The results of the Two-Way Mixed ANOVA on the effects of psychotic condition and frame condition showed that the assumption of sphericity was not violated, as assessed by Mauchly’s Test of Sphericity (*P* = 0.15). There was a significant main effect of frame condition on rod deviation errors *F*_(2, 30)_ = 6.71, *P* = 0.002, partial η^2^ = 0.18, with deviation errors in the tilted frame conditions (mean: Frame^–18°^ = 1.79; Frame^+18°^ = 1.80) being of greater magnitude from those for the Frame^0°^ condition (mean = 1.14). The results also show that there was a significant main effect of psychotic condition *F*_(1, 30)_ = 6.37, *P* = 0.02, partial η^2^ = 0.18, on absolute deviation errors, with SCZ generally displaying larger values (mean = 2.18) than healthy controls (mean = 1.37). There was no significant interaction between psychotic condition and frame condition *F*_(1, 30)_ = 1.59, *P* = 0.22, partial η^2^ = 0.10.

### Algebraic (Signed) error

SVV did not differ significantly (*t* = 0.78, *P* = 0. 44, df = 30) between controls (mean = −0.21 ± 0.94) and SCZ (mean = −0.51 ± 1.19).

Analysis by the Two-Way Mixed ANOVA on the effects of psychotic condition and frame condition showed that the assumption of sphericity was violated, as assessed by Mauchly’s Test of Sphericity (*P* < 0.001). Therefore, degrees of freedom were corrected using Greenhouse- Geisser estimates of sphericity (ε = 0.55). As expected, there was a significant main effect of frame condition on deviation errors *F*_(1.38, 30)_ = 32.38, *P* < 0.001, partial η^2^ = 0.52, with deviation errors in the tilted frame conditions (mean: Frame^–18°^ = −1.86; Frame^+18°^ = 0.97) of greater magnitude from those for the Frame^0°^ condition (mean = −0.34). There was no significant main effect of psychotic condition *F*_(1, 30)_ = 0.67, *P* = 0.42, partial η^2^ = 0.02, on signed deviation errors, with control (mean = −0.22) and SCZ (mean = −0.62) performing similarly overall. There was no significant interaction between psychotic condition and frame condition *F*_(1.38, 30)_ = 3.21, *P* = 0.07, partial η^2^ = 0.10.

For the derived measures, there was no significant difference between the two groups in RFE^–18°^ (*t* = 1.65, *P* = 0.11) nor RFE^+18°^ (*t* = 1.46, *P* = 0.12). However, σ°, the derived variability of error, was significantly greater in SCZ in comparison to controls (Welch’s *t* = 3.13, *P* = 0.006, df = 17) exceeding that for controls by 0.71°.

**FIGURE 4:**
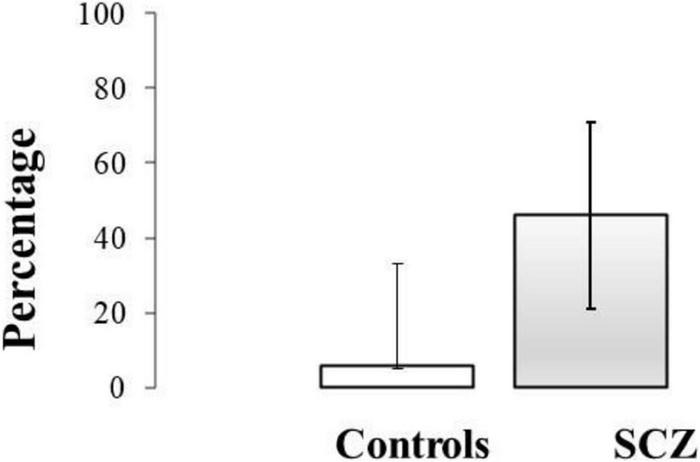
The 95% confidence intervals (CIs) for the proportion of control participants and SCZ with variability (σ°) greater than 2SD of the control mean value.

Data for σ° from the healthy group were used to establish an upper limit for the reference range of control σ° values and was calculated as Mean + 2SD. As mean σ° for the healthy group was 0.92 ± 0.27°, this upper limit equated to 1.46°. Only one healthy participant exceeded this upper limit value, and seven SCZ were outside of this range. Mean σ° for SCZ falling into this category was 2.41 ± 0.58°. The 95% confidence intervals (CIs) for the proportion of participants with errors greater than 2SD of the control were calculated using the Wilson method (Figure 4).

Despite there were no group differences in deviation error, we explored whether SCZ’s signed deviation errors or derived values were related to any clinical characteristics, specifically age at diagnosis, duration of disease, duration of inpatient stay, or PANSS scores. Spearman Rank Order correlation analyses were conducted between verticality measures and these clinical characteristics or PANSS scores (Table 3). There was no correlation between any signed deviation error/derived measure and any clinical characteristic, PANSS-P or PANSS-GP score, however, σ° correlated positively with PANSS-N (*P* = 0.02). Absolute Frame −18° also correlated positively with PANSS-N (*P* = 0.007).

**TABLE 3:** Correlation between SCZ clinical measures, psychotic symptoms severity and deviation errors or derived measures on the RFT.

	Age at diagnosis of disease	Illness duration	Inpatient stay	PANSS-N	PANSS-P	PANSS-GP
**Signed error**						
SVV	0.01 (0.97)	0.15 (0.59)	0.01 (0.99)	−0.11 (0.69)	0.12 (0.68)	0.02 (0.95)
Frame^0°^	0.27 (0.32)	0.23 (0.41)	−0.06 (0.82)	−0.23 (0.41)	0.31 (0.26)	−0.14 (0.64)
Frame^–18°^	−0.04 (0.88)	0.11 (0.70)	0.14 (0.63)	−0.51 (0.05)	−0.15 (0.60)	−0.10 (0.72)
Frame^+18°^	0.37 (0.17)	0.32 (0.10)	0.11 (0.70)	−0.06 (0.84)	0.05 (0.86)	−0.18 (0.53)
RFE^–18°^	−0.49 (0.07)	−0.03 (0.91)	0.41 (0.13)	−0.44 (0.10)	−0.41 (0.13)	−0.15 (0.59)
RFE^+18°^	0.31 (0.26)	0.29 (0.30)	0.27 (0.34)	−0.01 (0.96)	−0.02 (0.93)	0.04 (0.89)
σ°	0.31 (0.26)	0.04 (0.88)	−0.26 (0.34)	**0.58 (0.02)**	0.25 (0.38)	−0.38 (0.17)
Absolute error						
SVV	0.47 (0.07)	0.16 (0.57)	−0.18 (0.51)	0.36 (0.19)	0.11 (0.69)	−0.12 (0.68)
Frame ^0°^	0.16 (0.57)	0.39 (0.15)	0.24 (0.38)	0.38 (0.16)	0.15 (0.60)	−0.32 (0.24)
Frame ^–18°^	0.17 (0.54)	0.03 (0.92)	−0.19 (0.50)	**0.67 (0.007)**	0.23 (0.41)	−0.02 (0.95)
Frame ^+18°^	0.45 (0.09)	0.14 (0.62)	−0.11 (0.70)	0.06 (0.83)	0.38 (0.16)	0.02 (0.95)

Values are for Spearman Rank correlation coefficient and (P-Value), n = 15.

Significant correlations are in bold font.

PANSS, the positive and negative syndrome scale. “N” for negative symptoms, “P” for positive symptoms, “GP” for general psychopathology subscale.

**FIGURE 5:**
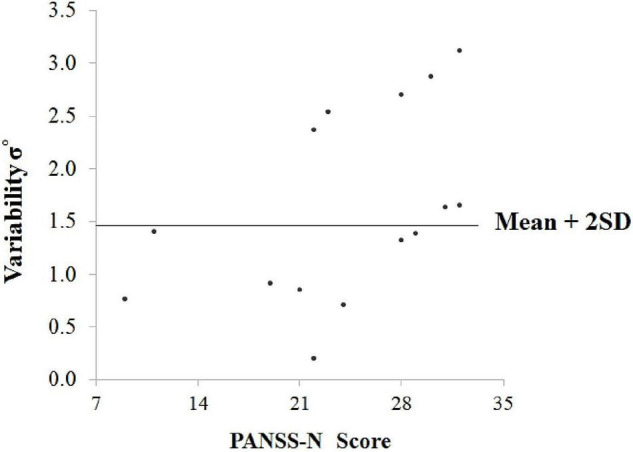
Scatterplot illustrating the relation between σ° (variability) and PANSS-N scores for SCZ. The previously calculated upper limit for the reference range of σ° values in controls (mean + 2SD) is also included in the graph.

Figure 5 is a scatterplot illustrating the relation between σ° and PANSS-N scores for SCZ. The previously calculated upper limit for the reference range of σ° values in controls (mean + 2SD) is also included in the graph. The subgroup of 7 SCZ participants who exceeded this upper limit had a mean PANSS-N score of 28.29 ± 4.19, significantly greater (*t* = 2.53, *P* = 0.03, df = 13) than that for the subgroup (*n* = 8) who did not exceed the calculated upper limit (mean = 20.38 ± 7.25). For these two subgroups, the corresponding mean PANSS-P score was 22.71 ± 7.20 and 19.25 ± 6.94, (and mean PANSS-GP score was 27.43 ± 8.40 and 30.88 ± 7.98, respectively, and not statistically different between the two subgroups (PANSS-P: *t* = 0.95, *P* = 0.36; PANSS-GP: *t* = 0.70, *P* = 0.49).

## Discussion

The present study was designed to test if there was a difference between healthy and patients with schizophrenia in the judgement of visual vertical in the presence of a surrounding tilted frame. Due to the adjustment method and precision of the recording system, we were able to employ different analyses of visual vertical data, including signed deviation, unsigned errors, the RFE and the intraindividual variability measure.

Our results indicate to lack of group difference in the no-context condition, which is identical to the main context condition except that no surrounding context was present, confirming that there were no baseline biases of participants, and verifying that participants understood and could perform the task. Analysis of the absolute (unsigned) error could distinguish the general performance on the RFT between healthy and SCZ, with SCZ generally displaying larger alignment deviations than healthy controls. However, the lack of interaction between psychotic and frame condition indicates that in no specific frame condition was there a significant group difference in rod deviation errors. The signed error and its derived measure RFE could not distinguish performance between the two groups. As such, our results indicate that that the overall strength of contextual modulation on the RFT did not differ significantly between controls and schizophrenia patients and agree with those by Shanker (9). Our results are also in line with those reported for other contextual modulation tasks (5, 15, 16), and do not support the hypothesis of a modified, and typically diminished, susceptibility to contextual modulation in schizophrenia patients (3–5).

The results however show that SCZ affects the uncertainty of the visual vertical upon contextual modulation. The increased intraindividual variability in rod alignment for SCZ during orientation modulation of visual vertical, in comparison to controls, was greater than the resolution of the recording system (0.50°) and may indicate a clinically functional difference. Increased variability in rod alignment on the RFT has previously been reported in studies on neck pain (17, 18), diabetes (19) and Parkinson’s disease (20), and may be a general indicator of neurological malfunction (21). Variability-related results in this study also reveal that there is a subgroup (about 50%) of SCZ who exceeded the upper limit for the reference range of control variability values (Mean + 2SD), having a mean greater by one degree than 2SD of the corresponding control value, indicating that SCZ who fell in this category were inconsistently aligning the rod relative to the frame.

In the current study, there was no association between SCZ clinical characteristics, such as illness duration, age at diagnosis, or inpatient stay, and any of the PANSS scores. However, there was a significant positive correlation (*r* = 0.58, *P* = 0.02) between PANSS-N scores and the variability measure in SCZ, indicating greater variability or inconsistency of rod alignment with more severe negative symptoms. Specifically, the subgroup of SCZ who exceeded the upper limit for the reference range of control variability values had a significantly greater mean PANSS-N score than the subgroup who did not exceed this upper limit. All these findings indicate that more severe negative symptoms for SCZ were associated with greater inconsistency in rod alignment. Several possible explanations for such finding may include a deficit in decisional resources, which is quite common in SCZ (7) or a subjective reduction in motivation or a state of avolition, a key negative symptom construct related to functional deterioration (11, 22, 23).

Another interesting result, which was identified for the leftward tilted (Frame ^–18°^), but not the rightward tilted frame (Frame^+18°^), is the significant correlation between PANSS-N scores with each of signed error (*r* = 0.51, *P* = 0.05) and absolute (unsigned) error (*r* = 0.67, *P* = 0.007). This indicates to greater contextual modulation of verticality perception when the sides of the frame were tilted to the left, as the negative symptoms became more severe in SCZ. The greater leftward bias in SCZ with more severe negative symptoms is consistent with studies reporting that leftward preference and severity of psychotic symptoms are correlated (24, 25). These findings may suggest to greater allocation of attention to the left side of the frame with more severe negative symptoms in SCZ.

The right cerebral hemisphere dominates in processing global features of visuospatial presentations (26, 27), and more frequently of the left side (28). There is also evidence that the laterality of visuospatial/orienting bias is under dopaminergic control in humans (29), with a preference for the visual space contralateral to the hemisphere with greater dopaminergic activity (30). Accordingly, for SCZ with more severe negative symptoms, the greater bias toward a frame tilted to the left side of space when processing the RFT may reflect a greater dominance and a more hyperdopaminergic activity in the right cerebral hemisphere (31) for this subgroup of patients, compared to those with less severe symptoms.

Finally, it would be interesting to evaluate performance on the RFT in unmedicated SCZ as antipsychotic treatment may influence visuospatial attention asymmetries in SCZ (32). As for other psychiatric illnesses, it would be particularly worthy to explore whether the observed leftward orientation bias on the RFT in SCZ with severe symptoms may be reversed in patients with clinical depression, as they are characterized by a left visuospatial deficit leading to right spatial bias (33–35).

There are a few limitations to this study, such as the small sample size that may have resulted in the moderate correlation effect sizes. Another limitation is not including SCZ with more severe negative symptoms (highest PANSS-N score in this study was 32), and it would be worthy to include in future studies, SCZ with higher PANSS-N scores and explore whether the observed correlations would be reinforced to a greater extent. Another limitation of our study was not determining the exact duration of psychosis or untreated psychosis, as well as not obtaining the chlorpromazine equivalent doses of the antipsychotic drugs that SCZ were receiving for treatment.

## Conclusion

Patients with schizophrenia and healthy controls were similarly affected by orientation contextual modulation of judging rod verticality. The intraindividual variability measure could distinguish between controls and SCZ, indicating that the adjustment method on a computerized RFT with high resolution is sensitive to detect subtle differences in verticality perception between healthy controls and SCZ. Despite the lack of group differences in raw deviation error data, PANSS-N depicted verticality perception upon contextual modulation with a leftward tilted frame, confirming previous findings of leftward preference for SCZ with more severe psychotic symptoms, and indicating to some degree of hemispheric asymmetry in orientation-related contextual modulation of visual vertical in this subgroup of schizophrenia patients. The increased variability in rod alignment for SCZ with more severe negative symptoms may be related to poor decision resources or generalized indifference in these patients.

## Data availability statement

The raw data supporting the conclusions of this article will be made available by the authors, without undue reservation.

## Ethics statement

The studies involving human participants were reviewed and approved by the Research and Ethics Committee at the College of Medicine and Medical Sciences at the Arabian Gulf University. The patients/participants provided their written informed consent to participate in this study.

## Author contributions

RR formulated the research proposal idea, provided training on the RFT software for data collection, and wrote the manuscript. HJ recruited the patients, provided the physical space for data collection, and edited the manuscript. MH helped in editing the manuscript. MA instructed the volunteers, monitored them, and recorded the RFT data while they performed the computerized RFT task. JB developed and provided the computerized RFT software. All authors contributed to the article and approved the submitted version.

## Conflict of interest

The authors declare that the research was conducted in the absence of any commercial or financial relationships that could be construed as a potential conflict of interest.

## Publisher’s Note

All claims expressed in this article are solely those of the authors and do not necessarily represent those of their affiliated organizations, or those of the publisher, the editors and the reviewers. Any product that may be evaluated in this article, or claim that may be made by its manufacturer, is not guaranteed or endorsed by the publisher.
